# Pigmentation effects of blue light irradiation on skin and how to protect against them

**DOI:** 10.1111/ics.12637

**Published:** 2020-07-20

**Authors:** R. Campiche, S. J. Curpen, V. Lutchmanen‐Kolanthan, S. Gougeon, M. Cherel, G. Laurent, M. Gempeler, R. Schuetz

**Affiliations:** ^1^ DSM Nutritional Products Personal Care & Aroma Wurmisweg 576 Kaiseraugst 4303 Switzerland; ^2^ Centre International de Développement Pharmaceutique (CIDP) BioPark Mauritius SOCOTA Phoenicia Sayed Hossen Road Phoenix 73408 Mauritius; ^3^ Newtone Technologies 13 bis Place Jules Ferry Lyon 69006 France

**Keywords:** Blue light, hyperpigmentation, niacinamide, *Scenedesmus rubescens*, skin

## Abstract

**Background:**

Visible light, in particular blue light, has been identified as an additional contributor to cutaneous photoageing. However, clinical studies demonstrating the clear effect of blue light on photoageing are still scarce, and so far, most studies have focused on broad‐spectrum visible light. Although there is evidence for increased skin pigmentation, the underlying mechanisms of photoageing *in vivo* are still unclear. Furthermore, there is still a need for active ingredients to significantly protect against blue light‐induced hyperpigmentation *in vivo*.

Our study had two aims: to detect visible changes in skin pigmentation following repeated irradiation of the skin with LED‐based blue light and to reduce pigmentation using suitable active ingredients.

**Method:**

We conducted a randomized, double‐blind and placebo‐controlled clinical study on 33 female volunteers with skin phototypes III and IV. We used a repetitive blue light (4 × 60 J cm^−2^, 450 nm) irradiation protocol on the volunteers’ inner forearms. Using hyperspectral imaging, we assessed chromophore status. In addition, we took chromameter measurements and photographs to assess visible hyperpigmentation.

**Results:**

We measured significant changes in chromophore status (*P* < 0.001 vs baseline), that is of melanin, haemoglobin and oxygen saturation, immediately after blue light irradiation. In addition, we found visible skin colour changes which were expressed by a significant decrease in ITA° values (delta ITA° = −16.89, *P* < 0.001 vs baseline for the placebo group) and an increase in a* (delta a* = +3.37, *P* < 0.001 vs baseline for the placebo group) 24 h post‐irradiation. Hyperpigmentation and skin reddening were mitigated by both a formulation containing 3% of a microalgal product and a formulation containing 3% niacinamide.

**Conclusion:**

Our study sets out an efficient and robust protocol for investigating both blue light‐induced cutaneous alterations, such as changes in skin chromophores, and signs of photoageing, such as hyperpigmentation. Moreover, we have shown evidence that both an extract of the microalga *Scenedesmus rubescens* and niacinamide (vitamin B3) have the potential to protect against blue light‐induced hyperpigmentation.

## Graphical abstract

We provide here a method to study blue light induced changes in skin in vivo. We show that an algal extract and niacinamide can mitigate blue light induced visible pigmentation and skin reddening.

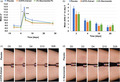

## Resume

**Contexte:**

La lumière visible, en particulier la lumière bleue, a été identifiée comme un facteur supplémentaire du photo‐vieillissement cutané. Cependant, les études cliniques, démontrant l’effet réel de la lumière bleue sur le photo‐vieillissement, sont encore rares et jusqu’à présent, la plupart des études portaient sur l’influence de la lumière visible à large spectre. Bien qu’il y ait des preuves concernant l’effet sur la pigmentation de peau, les mécanismes sous‐jacents du photo‐vieillissement in vivo sont encore peu clairs. De plus, le besoin d’ingrédients actifs protégeant de manière significative en in vivo contre l’hyperpigmentation induite par la lumière bleu est toujours présent.

**Notre étude a eu deux objectifs:**

Détecter des changements visibles dans la pigmentation de la peau à la suite d’une irradiation répétée avec de la lumière bleue à base de LED, et réduire la pigmentation à l’aide d’ingrédients actifs adaptés.

**Méthode:**

Nous avons mené une étude clinique randomisée, à l’aveugle et controlée avec un placebo sur 33 volontaires féminins de phototypes de peau III et IV. Nous avons défini un protocole d’irradiation répétitif à lumière bleue (4 x 60 J cm‐2, 450 nm) sur les avant‐bras intérieurs des volontaires. En utilisant l’imagerie hyperspectrale nous avons évalué l’état de chromophore. En outre, nous avons pris des mesures de couleur et des photographies pour évaluer l’hyperpigmentation de manière visuelle.

**Résultats:**

Nous avons mesuré des changements significatifs dans le statut de chromophore (p<0.001 par rapport au statut initial), par exemple au niveau de la mélanine, de l’hémoglobine et de la saturation en oxygène, immédiatement après l’irradiation à lumière bleue. De plus, nous avons constaté des changements visibles de couleur de la peau qui ont été exprimés par une diminution significative des valeurs ITA° (delta ITA° valeurs = ‐16.89, p<0.001 par rapport au statut initial pour le groupe placebo), et une augmentation de a* (delta a* = +3.37, p <0.001 par rapport au statut initial pour le groupe placebo) 24 heures après l’irradiation. L’hyperpigmentation et les rougeurs de la peau ont été atténués par une formulation contenant 3% d’un extrait d’algue ainsi que par une formulation contenant 3% de niacinamide.

**Conclusion:**

Notre étude a établi un protocole efficace et robuste pour étudier à la fois les altérations cutanées induites par la lumière bleue, telles que les changements dans les chromophores de la peau, ainsi que les signes de photo‐vieillissement, tels que l’hyperpigmentation. Enfin, nous avons prouvé qu’un extrait de l’algue Scenedesmus rubescens et la niacinamide (vitamine B3) avaient le potentiel de protéger contre l’hyperpigmentation induite par la lumière bleue.

## Introduction

Solar radiation, in particular ultraviolet radiation (UVR), is still considered to be the main cause of skin ageing, a phenomenon known as photoageing [1, 2]. However, for a couple of years now, visible light, with a wavelength of 400–700 nm and accounting for around 50% of all solar radiation, has come into focus as an additional contributor to photoageing. More specifically, high energy visible (HEV) light, commonly referred to as blue light – with a wavelength of 400–500 nm adjacent to UVA light – has been shown to induce signs of cutaneous photoageing *in vitro*, *ex vivo* and *in vivo* [3, 4].

The established effects of blue light on skin include oxidative stress and increased pigmentation. Various studies have demonstrated reactive oxygen species (ROS) formation upon irradiation with visible light. For example, it has been shown that irradiation of HaCaT keratinocytes with 41.35 J cm^‐2^ blue light of 453 nm wavelength leads to a rapid increase of ROS after 1 h [5]. Earlier studies have found that blue light induces oxidative stress via photoreduction of intracellular flavins [6] and that normal human keratinocytes show a rapid increase of intracellular ROS upon blue light irradiation [7]. Furthermore, Liebel *et al*. have used visible light to induce ROS in a dose‐dependent manner, pro‐inflammatory cytokines, such as IL‐1 and IL‐6, and matrix metalloproteases (MMPs) 2 and 9 in human skin equivalents. In addition, they have shown that anti‐oxidants, such as gamma‐tocopherol and a plant extract [8] suppress these markers. However, the influence of blue light on inflammation is controversial as other studies have found no modulation of inflammatory markers in keratinocytes irradiated with blue light between 412 and 453 nm [9]. A very recent study showed that in dermal fibroblasts irradiated with 150 J cm^‐2^ of various wavelengths, ROS formation was detected from 400 nm to 500 nm but no longer at 582 nm [10].

Skin has established its own defence system against oxidative stress by storing potent anti‐oxidants such as glutathione and carotenoids. Irradiation of human skin *in vivo* using 100 J cm^‐2^ blue‐violet light of 380–95 nm results in a significant decrease in cutaneous carotenoids as measured by Raman spectroscopy, suggesting the formation of free radicals [11]. Oxidative stress can have many causes in tissue, one of which is the formation of protein carbonyls, a process which is triggered in particular upon UVA irradiation [12]. Furthermore, in a recent *ex vivo* study on skin explants irradiated with 100 J cm^‐2^ high‐energy visible light with a peak wavelength of 420 nm, we found increased protein carbonylation [13].

Photo‐oxidation of melanogenic precursors may lead to so‐called immediate pigment darkening (IPD) and persistent pigment darkening (PPD) induced by irradiation of skin with UVA or visible light [14]. IPD is characterized by a greyish darkening observed immediately after irradiation and fading shortly afterwards, whereas a brownish‐black pigmentation can develop over several weeks in PPD without the involvement of melanogenesis processes.

Today, it is well established that irradiation with blue light causes hyperpigmentation in skin [15]. A consequence of this can be mottled hyperpigmentation, which is a visible sign of photoageing [16, 17], or age spots [18]. Mahmoud *et al*. found that visible light‐induced skin pigmentation was more sustained than pigmentation induced by UVA radiation [19]. In addition, this effect was seen in skin phototypes IV to VI, but not in phototype II [19]. On a mechanistic level, there is evidence that visible light (400–700 nm) induces melanin deposition in skin explants [20]. This particular study found that both gene‐expression and tyrosinase activity were increased by visible light *ex vivo*, suggesting activation of melanogenesis [20]. It also found that PPD, lasting for up to ten days after exposure, could only be induced by repeated irradiation with visible light *in vivo* on human skin phototypes V and VI, but that pigmentation faded after 24 h when only a single irradiation pulse was used. More recently, irradiation of human skin with visible and near infrared has been shown to trigger changes in stratum corneum lipids, suggesting that visible light has an impact on skin barrier function [21].

Concerning protection against visible light‐induced cutaneous changes, and hence photoageing, it has been stated that additional means to UV filters are needed [22, 23]. For example, Liebel *et al*. found that protection against UV radiation (UVR) and visible light irradiation was only fully achieved when a combination of anti‐oxidants was used in addition to UVA and UVB filters [8]. Others have proposed a visible light protection factor for sunscreens containing mineral UV filters such as titanium dioxide or iron oxide. This suggests that sunscreens with UV filters do not offer sufficient protection against solar radiation‐induced cutaneous photoageing. This is also the case if sunscreens contain physical filters such as titanium dioxide (TiO_2_), which scatters light in the visible spectrum, and methylene bis‐benzotriazolyl tetramethylbutylphenol (MBBT) [24, 25]. Moreover, adsorbents in the visible spectrum could have the disadvantage that they are indeed visible, meaning they could give the skin a yellow to reddish tone which is normally less preferred by consumers. Thus, alternative means of protection from visible light, for example by anti‐oxidants or pigmentation inhibitors, are needed.

In this study, we wanted to investigate *in vivo* the effects of blue light irradiation, and how to prevent them, in human skin phototypes III and IV. We focused on pigment darkening and investigated skin chromophore as well as skin colour changes. We used an LED lamp with a single emission peak of around 450 nm, which corresponds to deep blue light as it is emitted from both the sun and from electronic devices. We conducted a double‐blind, placebo‐controlled and randomized study using niacinamide (vitamin B3) and an extract of the green freshwater microalga *Scenedesmus rubescens*. Both niacinamide and the microalgal extract have previously shown protective effects against cutaneous signs of photoageing induced by solar radiation [13, 26].

## Material and methods

### Test compounds

We used a base formulation (placebo) and two active formulations consisting of the base formulation plus 3% niacinamide (commercial product Niacinamide PC, DSM Nutritional Products, Switzerland), and 3% of a commercial product containing 2.5% dry extract of the green freshwater microalga *Scenedesmus rubescens* (trade name PEPHA^®^‐AGE, DSM Nutritional Products, Switzerland), respectively. Base and active formulations are outlined in Table 1.

**Table 1:** Formulations used in this study

	Product A (Placebo)	Product B (with microalgae extract)	Product C (with niacinamide)
INCI Name	%	%	%
Aqua	67.24	64.24	64.20
Sodium gluconate	0.20	0.20	0.20
Propanediol	5.00	5.00	5.00
Xanthan gum	0.20	0.20	0.20
Cetearyl olivate; sorbitan olivate	4.00	4.00	4.00
Cetearyl alcohol	1.50	1.50	1.50
Phenoxyethanol; ethylhexylglycerin	1.00	1.00	1.00
Caprylic/capric triglyceride	8.00	8.00	8.00
Octyldodecanol	10.00	10.00	10.00
Dimethicone	2.00	2.00	2.00
Hydroxyethyl acrylate/sodium acryloyldimethyl taurate copolymer	0.40	0.40	0.40
Scenedesmus rubescens extract; aqua; phenoxyethanol	0.00	3.00	0.00
Niacinamide	0.00	0.00	3.00
Citric acid; aqua	0.16	0.16	0.20
Parfum	0.30	0.30	0.30
Total	100.00	100.00	100.00

### Blue light source

**Figure 1:**
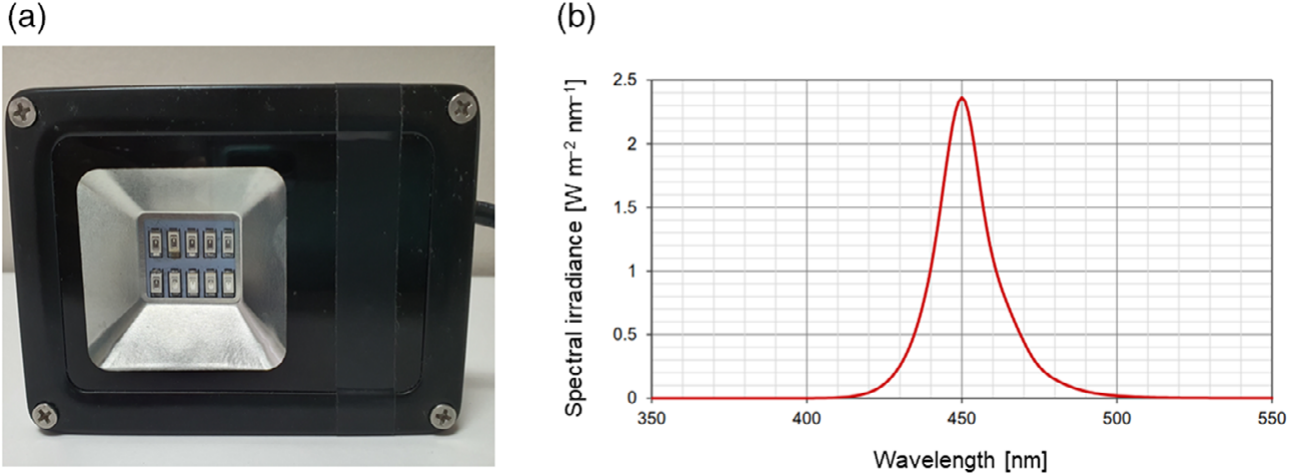
(a) Bottom view of the blue light lamp showing the assembly of 10 LEDs. (b) Spectral range of the blue light lamp with a single peak of around 450 nm.

The blue light lamp consisted of an assembly of 10 identical LEDs (light‐emitting diodes) (Honglitronic, Guangzhou, PRC) emitting continuous visible radiation embedded in a reflector and covered by a transparent glass window (Fig. 1a). The lamp’s emission characteristics were 54 W m^‐2^ irradiance at 9 cm distance, 2.1 mW power, single peak with a maximum wavelength of 450 nm and spectral range of approximately 420–500 nm (Fig. 1b). The aperture on the light source was 4.5 cm × 4.5 cm. The array at the surface of exposure was approximately 10 cm × 10 cm at an approximate distance of 5 cm from the light source. A Gentec EO Thermopile Detector was used to measure the precise intensity of the light source in Watt cm^‐2^ at the level of the investigational site. The time of exposure was adjusted to ensure that 60 J cm^‐2^ of blue light was delivered to the investigational site.

### Clinical study design

This was a placebo‐controlled, double‐blind and randomized clinical study conducted by the Centre International de Développement Pharmaceutique (CIDP) in Mauritius. Volunteers gave their informed consent to participate in the study, and the general principles of the Declaration of Helsinki guidelines were applied. The study was approved by the local ethics committee, Services Gestion des Compagnies Ltée – Comité d’éthique, BioPark Mauritius, Socota Phoenicia, Sayed Hossen Road, Phoenix 73408, Mauritius, under the project identification number EC18‐COS‐022. Adverse effects were recorded.

Thirty‐three healthy female volunteers aged 21 to 41 were recruited. The group consisted of 18 Caucasians, 6 Asians and 9 mixed‐ethnicity volunteers. 18 volunteers were skin phototype III, and 15 volunteers were skin phototype IV according to the Fitzpatrick score, and as assessed on the face. Two of the inclusion criteria were a commitment to refrain from sun exposure during the study, and that volunteers had an indoor, office, day job. Volunteers came to the study site having applied no products to the forearms. They acclimatized for 15 min in a room at 24 ± 2°C. One irradiation zone of 4 cm × 6 cm was identified and marked with positioning masks on the inner side of each forearm.

The study consisted of three phases: conditioning phase (D‐6 to D‐1), irradiation phase (D0 to D3) and recovery phase (D4 to D28). A standard daily dose of 2 mg cm^‐2^ of the respective formulation was applied to the volunteers’ forearms for the entire study except during irradiation phase (D0 to D3). Blue light irradiation took place on days 0, 1, 2 and 3, in the form of a dose of 60 J cm^‐2^ blue light with a single peak emission of around 450 nm. Hyperspectral images using the SpectraCam^®^ device (Newtone Technologies, Lyon, France) [27] were taken at days −6, 0 (immediately before irradiation), 3 (immediately after irradiation), 4, 10 and 28, and melanin content, haemoglobin content and oxygen saturation rate (ratio between oxygenated haemoglobin and total haemoglobin) were calculated. Images of the irradiated zones were taken the same way using a Nikon D7000 camera in the presence of a 48‐patch colour chart (Newtone Technologies, Lyon, France) to correct possible lighting variations. Similarly, measurements using a Chromameter^®^ (Minolta CR400, Konica Minolta, Tokyo, Japan) were taken in the middle of the exposed zones. The recorded L*a*b* values (CIELab colour system) were used to calculate individual topology angles ITA° using the formula ITA°=(arctan((L* − 50)/b*)) × 180/π [28], as well as ∆*E* using the formula ∆*E*_D0‐D28_ = √((L*_D0_ − L*_D28_)^2^ + (a*_D0_ − a*_D28_)^2^+(b*_D0_ − b*_D28_)^2^).

### Statistical analysis

Measurement samples were tested for normal distribution using the Shapiro–Wilk test. Three or more samples were analysed by one way ANOVA. The Student’s *t*‐test for unpaired or paired samples was used to test for significant differences between samples and versus the baseline. In case one or more samples were not normally distributed, the Mann–Whitney *U* test for unpaired samples was also employed. Error bars in graphs represent the standard error of the mean.

## Results

### Blue light‐induced changes in skin chromophores

**Figure 2:**
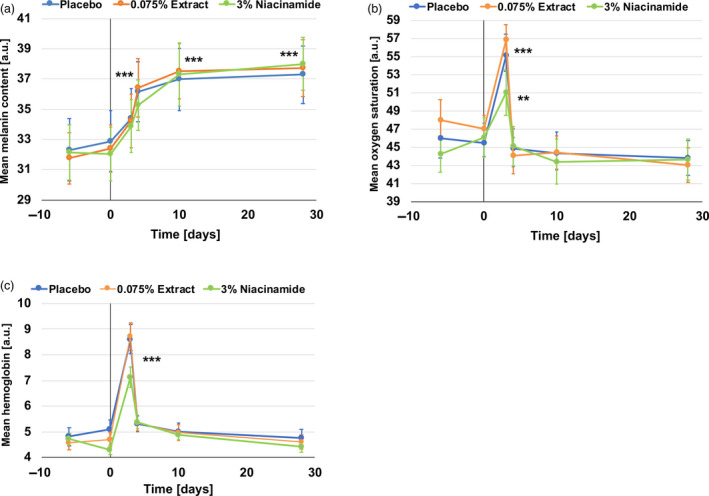
Chromophore content in skin measured by hyperspectral imaging. (a) Mean melanin content. An increase in melanin content is measured following blue light irradiation. (b) Mean oxygen saturation. A peak in increased oxygen saturation is measured immediately after the blue light irradiation phase. (c) Mean haemoglobin content. A peak in increased haemoglobin content is measured immediately after the blue light irradiation phase. ***P < 0.001, **P < 0.01 all vs day 0.

Using hyperspectral imaging provided by the SpectraCam^®^ device, we assessed melanin and haemoglobin content and oxygen saturation after blue light irradiation and during the subsequent recovery phase. We found a continuous increase in melanin following blue light irradiation; this reached significance one day after the blue light irradiation protocol ended. Melanin content remained constant until day 28 and was similar with all three formulations used (Fig. 2a). In addition, both oxygen saturation and haemoglobin measurements were up‐regulated significantly at day 3 immediately after blue light irradiation (Fig. 2b and c), with a trend for lower up‐regulation of haemoglobin in the niacinamide group (*P* = 0.096 vs placebo). However, one day after blue light irradiation at day 4 these values were back down to pre‐irradiation values.

### Blue light‐induced changes in skin colour

**Figure 3:**
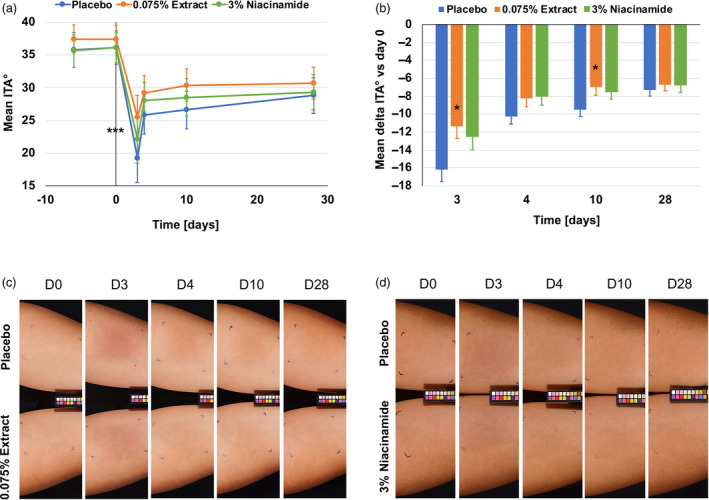
Blue light irradiation induced colour change and darkening of the skin and partial protection by the algal extract and niacinamide. (a) Mean ITA° decreased significantly in all three groups during the irradiation phase, from day 0 to day 3. During the recovery phase, from day 4 to day 28, the skin recovered mostly until day 10 and was stable until day 28. ***P < 0.001 vs baseline. (b) Mean delta ITA° values show significant protection by the algal extract compared to the placebo at days 3 and 10. After day 10, 7 days after irradiation, no difference was measured anymore. *P < 0.05 vs placebo. (c) Volunteer #12 with lighter skin using the placebo formulation on one arm and the algal extract formulation on the other arm is shown here. The difference in skin colour between the two formulations can be seen from day 3 to 10. (d) Volunteer #20 with darker skin using the placebo formulation on one arm and the niacinamide formulation on the other arm is shown here. The difference in skin colour between the two formulations can be seen from day 3 to day 10.

Using a Chromameter^®^ CR400 device we measured skin colour changes at various time points during the study. We found a significant decrease in ITA° value immediately after the irradiation phase at day 3. In the placebo group, we found a decrease in the mean ITA° from 36.11 to 19.32 (*P* < 0.001), suggesting visible hyperpigmentation. This decrease in ITA° was lower for the niacinamide and the microalgae groups: a drop from 36.07 to 22.11 and from 37.39 to 25.53 respectively (Fig. 3a). Although hyperpigmentation recovered during the recovery phase, it stayed significantly below the day 0 baseline. If we look at the delta ITA° values, there was a significant difference between the placebo and the microalgae group (*P* < 0.05) at day 3, immediately after irradiation, and at day 10, one week after irradiation (Fig. 3b), and a trend between the placebo and the niacinamide group immediately afterwards, until one week post‐irradiation with *P* = 0.071 at day 3, *P* = 0.078 at day 4, and *P* = 0.087 at day 10 (Fig. 3b).

**Figure 4:**
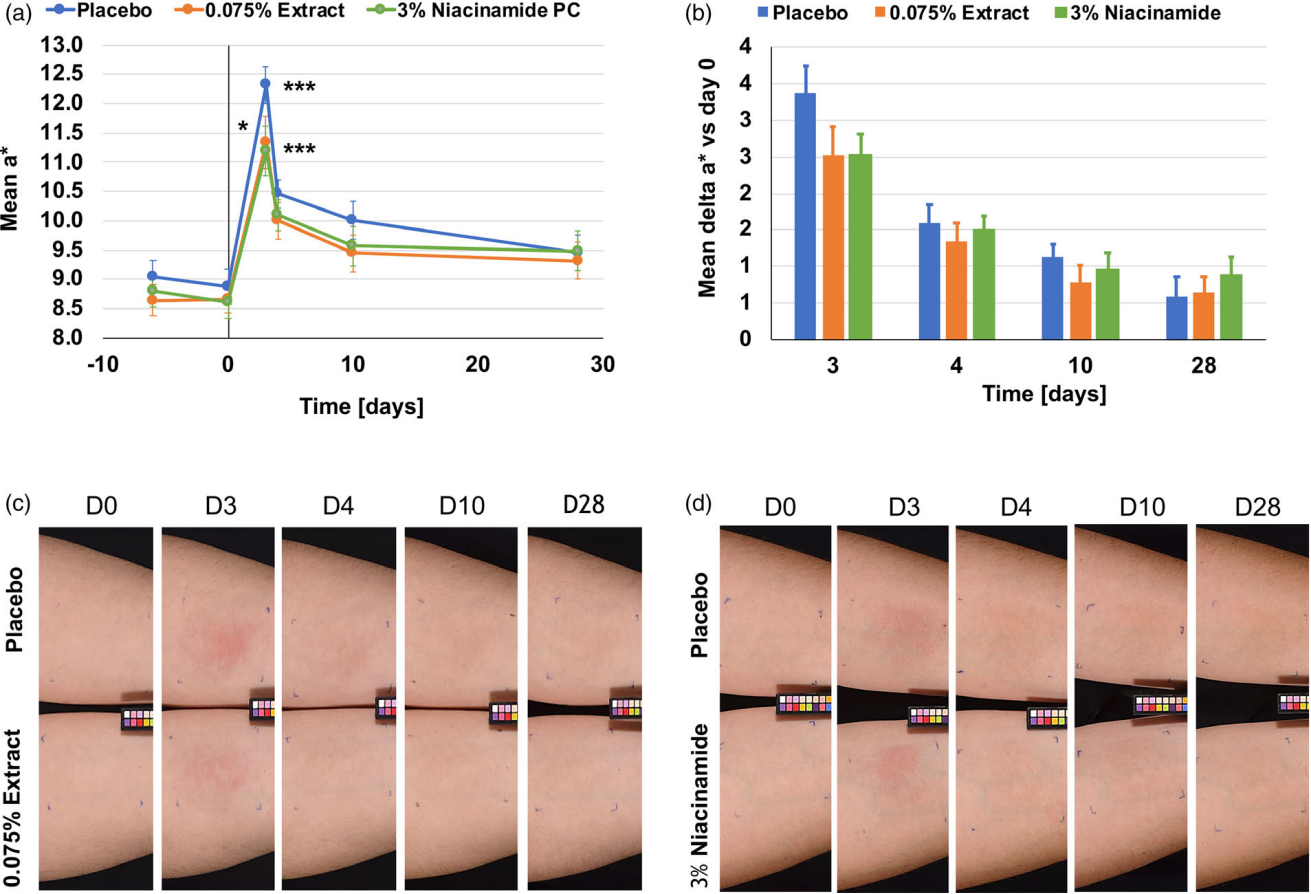
Blue light induces skin reddening. (a) Blue light irradiation a* values as measured on day 3. ***P < 0.001 vs day 0 for all groups. Compared to placebo, these values were significantly lower for niacinamide (*P < 0.05 niacinamide vs placebo). (b) Mean delta a* values show non‐significantly lower a* values for the algal extract and niacinamide compared to the placebo at days 3 to 10. (c) Volunteer #13 with lighter skin using the placebo formulation on one arm and the algal extract formulation on the other arm is shown here. The difference in skin reddening between the two formulations can be seen from day 3 to day 10. (d) Volunteer #16 with lighter skin using the placebo formulation on one arm and the niacinamide formulation on the other arm is shown here. The difference in skin reddening between the two formulations can be seen from day 3 to day 10.

Skin darkening was also indicated in the measured L* values which decreased significantly for all three groups during the irradiation period (Fig. S1). These measured results were also perceived visually. In Fig. 3c, we show a representative volunteer with skin hyperpigmentation induced by blue light irradiation. The protective effect of the microalgal formulation was also visible at days 3 to 10. In addition, skin reddening was increased by blue light irradiation, as measured by significantly increased a* values for all groups (*P < 0.001 day 3 vs day 0; Fig. 4a). Both formulations containing niacinamide (*P* < 0.05 vs placebo) and the microalgal extract (*P* = 0.079 vs placebo) showed protection against skin reddening immediately after blue light irradiation (Fig. 4a). This protective effect was also reflected in the mean delta a* compared to the baseline, showing a trend for niacinamide against placebo at day 3 (*P* = 0.082; Fig. 4b). This reddening effect was sometimes visible instead of the darkening effect, and we show here an example of a subject with the placebo formulation on one forearm and the microalgal formulation on the other forearm (Fig. 4c) where there was skin reddening rather than darkening.

**Figure 5:**
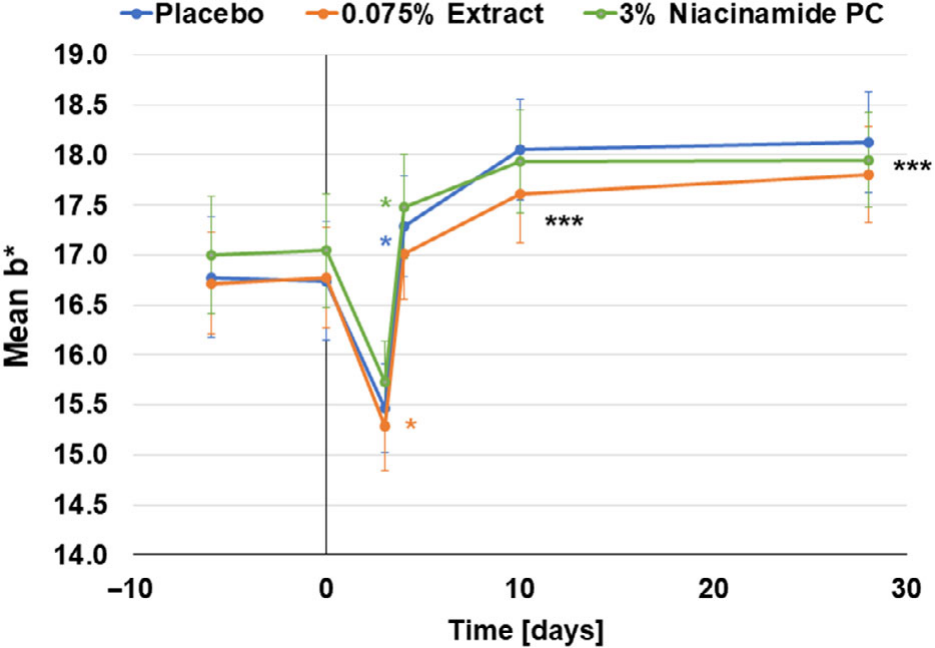
Blue light modulates b*‐values. After a drop in b* value after blue light irradiation, the values increase significantly above baseline day 0 values. ***P < 0.001, **P < 0.01, *P < 0.05, all vs day 0.

Finally, there was also a change in the b* value. Interestingly, it dropped immediately after blue light irradiation, but increased above the baseline level during the recovery phase (Fig. 5). The change in skin colour seen with L*, a* and b* was also reflected in Δ *E* values. The group using the microalgae formulation had a significantly smaller Δ *E* than the placebo group (*P* < 0.05) at day 3 (Fig. S2).

## Discussion

Here, we describe a new *in vivo* method for the measurement and analysis of blue light‐induced changes in skin parameters. This method employs an irradiation wavelength with a single peak of around 450 nm which is in the blue spectrum of solar light. It is thus different from previously reported similar methods which employed a broader range of visible light [19, 20, 29] or blue/violet light [15]. In addition, we irradiated skin with 60 J cm^‐2^ blue light on four consecutive days, accumulating approximately 240 J cm^‐2^. This 60 J cm^‐2^ dose can be obtained on a clear summer day in central Europe at around midday for about one hour. In line with previously reported studies, we also found clear and significant blue light irradiation induced colour change (Fig. 3). Interestingly, skin recovered rapidly during the first 24 h after irradiation but did not return to the baseline for the remaining period of the study. This would support previous reports that blue light‐induces persistent pigment darkening [14, 19, 20] similar to UVA [19]. Moreover, we continued this study for an additional month and again, a complete recovery of the skin was not observed (not shown). An increase in the a* parameter immediately after the irradiation phase was also perceived visually as the images in Figs. 3c and 4c show. Interestingly, the volunteer in Fig. 3 shows a tanning effect at day 4 and beyond, whereas the volunteer in Fig. 4 seems to primarily show a reddening effect whereas the tanning effect is less obvious. The volunteer in Fig. 3 had an ITA° of 35 at day 0 whereas the volunteer in Fig. 4 had an ITA° of 48 at day 0, indicating lighter skin. Looking at all the subjects with an ITA° of <41 (intermediate, brown phototype [28]) and those with an ITA° of >41 (light, very light phototype [28]), we found delta a* values of 2.56 and 3.23 (*P* = 0.353), respectively (Table S1). The same was seen when grouping the volunteers in phototype IV and III, with delta a* values of 2.52 and 3.17 (*P* = 0.173), respectively (Table S1). This might help explain the visible effect and support earlier findings suggesting that darker skin types tan more easily after visible light irradiation [19]. In addition, erythema, which is characterized by skin reddening, was deemed to occur more easily in lighter pigmented skin types than in darker ones and this was independent of wavelength, at least as investigated in the UV range [30]. However, there may be a dual effect such that pigmented skin is indeed less prone to erythema, because of melanin content, than non‐pigmented skin, and there the reddening effect is less camouflaged than in pigmented skin. The visible tanning effect found here was supported by measuring melanin using hyperspectral imaging (Fig. 2a). Again, melanin measurements stayed at an increased level after the irradiation phase and throughout the recovery phase. As melanin content increased from day 3 to day 4 and even as far as day 10, we speculate that melanogenesis did indeed occur. It has been shown before that visible light is able to induce markers of melanogenesis such as tyrosinase and to deposit melanin in human skin [20]. Kollias *et al*. proposed that the skin colour changes they found following visible light irradiation were because of photooxidation of melanin or other chromophores, rather than melanogenesis itself [14]. Regarding haemoglobin and oxygen saturation (Fig. 2b and c), we found a sharp increase during blue light irradiation which decreased again 24 h after irradiation was stopped. These measurements correspond with the visible effects of skin reddening seen primarily in the volunteer with the lighter skin in Fig. 4, but also, to a lesser extent, in the volunteer in Fig. 3. These are similar to findings previously reported by Mahmoud *et al*. who observed a dose‐dependent increase in oxy‐haemoglobin after visible light irradiation, which resolved below erythemal concentrations 2–24 h after irradiation [19]. We assume that the drop in the b* parameter observed in our study immediately after blue light irradiation may be because of the increase in the a* parameter; haemoglobin and oxygen saturation giving the skin a bluish to reddish appearance. When the skin reddening effect faded, the yellowish appearance of the skin was more visible, reflecting the increase in the b* parameter and suggesting photoageing phenomena such as protein carbonylation [13] and other protein oxidation processes [31]. Along these lines, oxidative stress was shown to be induced by visible light [8] and blue light [10, 13] and such events can lead to accumulations of extracellular matrix components such as lipofuscin, leading to ageing‐dependent changes in skin pigmentation [31] independent of melanogenesis.

Finally, we investigated the effects of a microalgal extract and vitamin B3 (niacinamide) on blue light irradiated skin. We mainly found a protective action against visible changes in skin colour (Figs 3, 4 and Supplementary Material). However, no changes were measured in the chromophores analysed (Fig. 2). Microalgal extracts such as the genus *Scenedesmus* are rich in polyphenols and have been shown previously to have anti‐oxidative potential [32, 33]. We ourselves could show that the extract used here had an anti‐oxidative action, *ex vivo*, against blue light‐induced protein carbonylation and ROS formation [13]. We also showed that the extract was able to protect skin from UVR induced collagen III degradation [26]. This contributes to the skin’s increased resistance to blue light‐induced pigmentation events independent of melanogenesis. In addition, niacinamide is also an anti‐oxidant [34, 35] and is well known for working against ageing‐induced mottled hyperpigmentation [36, 37]. As such, it supports to mitigate blue light‐induced photoageing such as the hyperpigmentation shown here (Fig. 3a). Thus, we propose that both an extract of *Scenedesmus rubescens* and niacinamide could be valuable additions to sunscreen formulations to protect skin against solar irradiation. It has been shown previously that UV filters are not able to fully protect skin against irradiation in the visible spectrum [8, 10, 13]. However, when anti‐oxidants were added to the formulation, protection is significantly improved [8, 10, 13].

## Conclusions

We present a new method for investigating the effects of blue light on skin *in vivo*. By this method, significant modulation of parameters like melanin and haemoglobin content, skin oxygen saturation, and visible hyperpigmentation and erythema was measured. We found that an extract of the microalga *Scenedesmus rubescens* was able to significantly reduce visible hyperpigmentation in the first 10 days after irradiation with blue light, whereas a protective effect on skin reddening was found for niacinamide right after irradiation at day 3. This test set‐up allows for in vivo screening for appropriate skin care actives and UV filters to build claims for blue light protection.

## Conflicts of interest

This study was funded by DSM Nutritional Products. RC, GL, MG and RS are employees of DSM Nutritional Products and receive regular salaries from the company. The extract of the microalga *Scenedesmus rubescens* and niacinamide mentioned in this manuscript are marketed by DSM Nutritional Products under the trade names PEPHA^®^‐AGE and Niacinamide PC, respectively. The authors report no other conflicts of interest.

## Supporting information


**Figure S1**. Skin hyperpigmentation after blue light irradiation was also reflected in lower L* values at day 3 and beyond.
**Figure S2**. Skin color change after blue light irradiation was suppressed by the formulation containing the algal extract and niacinamide.
**Table S1**. Skin reddening effect in subjects using the placebo formulation and grouped by skin phototype.
