# Supplementary material for: Pigmentation effects of blue light irradiation on skin and how to protect against them
Source: Int J Cosmet Sci. 2020 Jul 20;42(4):399–406. doi: 10.1111/ics.12637 (PMC7496068; doi:10.1111/ics.12637)
Supplement: Supplementary file 1 — Figure S1. Skin hyperpigmentation after blue light irradiation was also reflected in lower L* values at day 3 and beyond. Figure S2. Skin color change after blue light irradiation was suppressed by the formulation containing the algal extract and niacinamide. Table S1. Skin reddening effect in subjects using the placebo formulation and grouped by skin phototype. [file ICS-42-399-s001.docx]

**Supplemental Material**


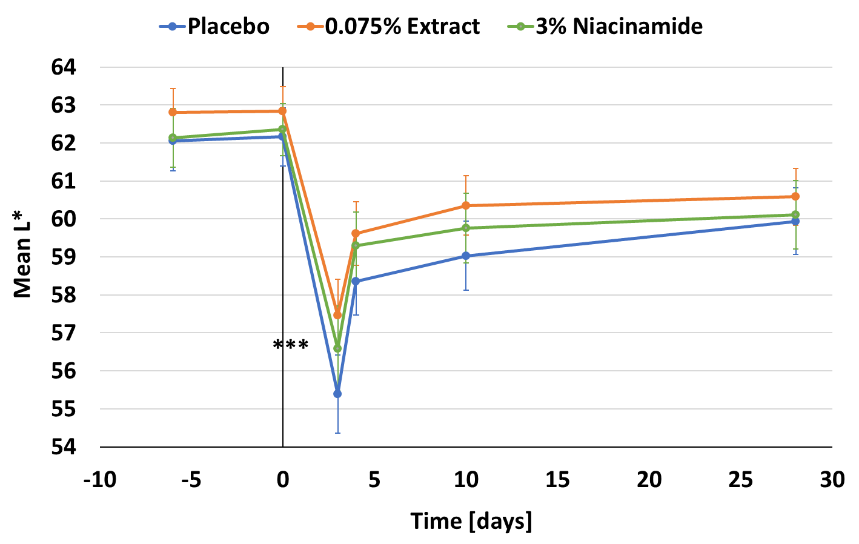


Figure S1: Skin hyperpigmentation after blue light irradiation was also reflected in lower L* values at day 3 and beyond. ***p<0.001 vs baseline day 0 for all three groups.


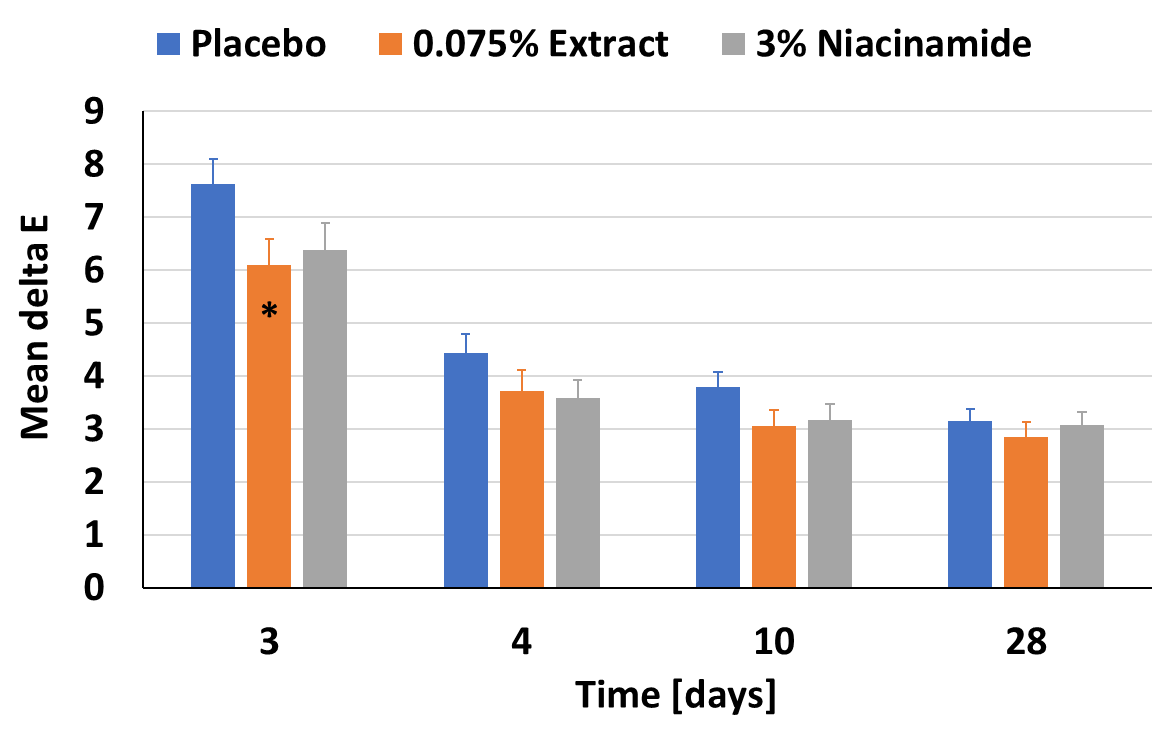


Figure S2: Skin color change after blue light irradiation was suppressed by the formulation containing the algal extract and niacinamide. *p<0.05, ^~~§~~^~~p<0.1 both~~ vs placebo formulation.

Table S1: Skin reddening effect in subjects using the placebo formulation and grouped by skin phototype.

|  | phototype | mean delta a* | STDV | SEM | p-value |
| --- | --- | --- | --- | --- | --- |
| subgroups | IV | 2.52 | 1.84 | 0.43 | 0.173 |
|  | III | 3.17 | 1.36 | 0.41 |  |
|  | ITA° < 41 | 2.56 | 1.62 | 0.36 | 0.353 |
|  | ITA° > 41 | 3.23 | 1.81 | 0.60 |  |
